# Effect of Long-Term Cisplatin Exposure on the Proliferative Potential of Immortalized Renal Progenitor Cells

**DOI:** 10.3390/ijms252312553

**Published:** 2024-11-22

**Authors:** Eloho Ighofose, Scott H. Garrett, Sarmad Al-Marsoummi, Aaron A. Mehus, Donald A. Sens, Sandeep K. Singhal, Sonalika Singhal, Seema Somji

**Affiliations:** Department of Pathology, School of Medicine and Health Sciences, University of North Dakota, Stop 9037, Grand Forks, ND 58203, USA; eloho.ighofose@und.edu (E.I.); scott.garrett@und.edu (S.H.G.); sarmad.al.marsoummi@und.edu (S.A.-M.); aaron.mehus@und.edu (A.A.M.); donald.sens@und.edu (D.A.S.); sandeep.singhal@und.edu (S.K.S.); sonalika.singhal@und.edu (S.S.)

**Keywords:** HRTPT, renal progenitor cells, cisplatin, proximal tubule, cell cycle

## Abstract

Cisplatin (CisPt) is a widely used chemotherapeutic agent. However, its nephrotoxic effects pose significant risks, particularly for the development of acute kidney injury (AKI) and potential progression to chronic kidney disease (CKD). The present study investigates the impact of non-lethal exposure of CisPt to immortalized human renal epithelial precursor TERT cells (HRTPT cells) that co-express PROM1 and CD24, markers characteristic of renal progenitor cells. Over eight serial passages, HRTPT cells were exposed to 1.5 µM CisPt, leading to an initial growth arrest, followed by a gradual recovery of proliferative capacity. Despite maintaining intracellular platinum (Pt) levels, the cells exhibited normal morphology by passage eight (P8), with elevated expression of renal stress and damage markers. However, the ability to form domes was not restored. RNA-seq analysis revealed 516 differentially expressed genes between CisPt-exposed and control cells, with significant correlations to cell cycle and adaptive processes, as determined by the Reactome, DAVID, and Panther analysis programs. The progenitor cells treated with CisPt displayed no identity, or close identity, with cells of the normal human nephron. Additionally, several upregulated genes in P8 cells were linked to cancer cell lines, suggesting a complex interaction between CisPt exposure and cellular repair mechanisms. In conclusion, our study demonstrates that renal progenitor cells can recover from CisPt exposure and regain proliferative potential in the continued presence of both extracellular CisPt and intracellular Pt.

## 1. Introduction

Cisplatin (CisPt) a chemical compound with the formula cis-[Pt(NH_3_)_2_Cl_2_] and is used as a chemotherapeutic treatment in many cancers, which include sarcomas, small cell lung cancer, squamous cell carcinoma of the head and neck, ovarian cancer, lymphomas, bladder cancer, cervical cancer, and germ cell tumors [1,2]. Its value as a chemotherapeutic agent is illustrated by its high remission rate in testicular cancer, where treatment improved remission rates from 5–10% before its use to 75–85% [3]. While CisPt has many side effects, nephrotoxicity is the primary dose-limiting side effect, which causes major clinical concerns for patients treated with this medication [4]. Studies have shown that CisPt selectively accumulates in the proximal tubule by basolateral-to-apical transport [4,5]. It has been proposed that accumulated CisPt disrupts mitochondrial energetics and endoplasmic reticulum calcium homeostasis and stimulates the production of reactive oxygen species and pro-inflammatory cytokines [6]. This laboratory’s interest in the nephrotoxicity of CisPt involves the inherent ability of the renal nephron to undergo tubular repair [7,8,9]. This repair process has been shown to involve a rare and sparsely located population of cells that co-express PROM1 (prominin 1, also known as CD133) and CD24 [8,10,11]. These cells have been shown to express all the properties expected of a renal progenitor cell [8,10,11]. While the repair and regeneration ability has been confirmed for these cells as well as their origin within the kidneys, mechanistic studies have been difficult and limited due to the rarity and distribution of the cells.

This laboratory tested numerous renal cell cultures to determine if any of the cells co-expressed PROM1 and CD24. This search identified the putative primary human cultures of proximal tubule cells and the hTERT immortalized renal cells, RPTEC/TERT1, which are comprised of a mixed population of cells composed of 60% cells co-expressing PROM1 and CD24 and the remaining only expressing CD24 [12]. Cell sorting was used by the laboratory to isolate two new immortalized cell lines from the human proximal tubular cell line RPTEC/TERT1, one named human renal tubular precursor TERT (HRTPT), which co-expresses both PROM1 and CD24, and a second named human renal epithelial cell 24TERT (HRECT24T), which expresses CD24 but not PROM1 [12]. These cell lines maintained their expected expression of PROM1 and CD24 over extended serial passages in cell culture. The HRTPT cells expressed features expected of progenitor cells, including the ability to undergo adipogenic, neurogenic, and tubulogenic differentiation, the ability for self-renewal (spheroid formation in low attachment flasks), and differentiation into tubules when grown in Matrigel™ [12]. The HRECT24T displayed none of these features. In the present study, the HRTPT cell line was used to determine the effect of CisPt exposure on a cell capable of renal repair and regeneration. The initial goal of the study was to determine the effect of CisPt on cell growth and toxicity following short- (48 h) and long-term exposure of the cells (eight serial passages). The second goal was to perform “omic” analysis of the cells in the presence of CisPt for eight passages to determine the changes in gene expression and pathways in the HRTPT cells following exposure. A long-term time course was chosen since most studies employ short exposure to be assigned to a single cellular process. The overall goal was to demonstrate that the HRTPT cells can model the role of renal progenitor cells in repair and regeneration.

## 2. Results

### 2.1. Long Term Exposure of HRTPT Cells to CisPt

**Figure 1:**
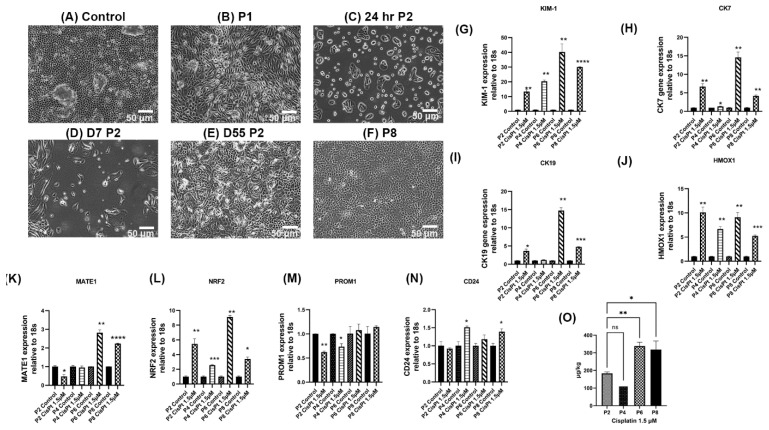
Long-term exposure of HRTPT to 1.5 µM CisPt. Confluent cultures of HRTPT cells were cultured in the presence of 1.5 µM CisPt for eight serial passages. (**A**–**F**) Morphology of the HRTPT cells as visualized by light microscopy. (**A**) Morphology of control cells. (**B**) Morphology of confluent cells at P1. (**C**) Morphology of HRTPT cells treated with 1.5 µM CisPt at P2 after 24 h. (**D**) Morphology of HRTPT cells treated with 1.5 µM CisPt at P2 after seven days. (**E**) Morphology of confluent HRTPT cells treated with 1.5 µM CisPt at P2 (D55). (**F**) Morphology of confluent HRTPT cells treated with 1.5 µM CisPt at P8. (**G**–**N**) Real-time qPCR analysis of the expression of *KIM-1* (**G**), *CK-7* (**H**), *CK19* (**I**), *HMOX1* (**J**), *MATE1* (**K**), *NRF2* (**L**), *PROM1* (**M**), and *CD24* (**N**). (**O**) Accumulation of Pt in HRTPT cells cultured in the presence of 1.5 µM CisPt for eight serial passages. ****; ***; **; * indicate significant differences in gene expression level compared to the control at *p*-value of ≤0.0001; ≤0.001; ≤0.01; ≤0.05, ns: *p* > 0.05, respectively. (**O**) Accumulation of Pt in HRTPT cells cultured in the presence of 1.5 µM CisPt for eight serial passages.

**Figure 2:**
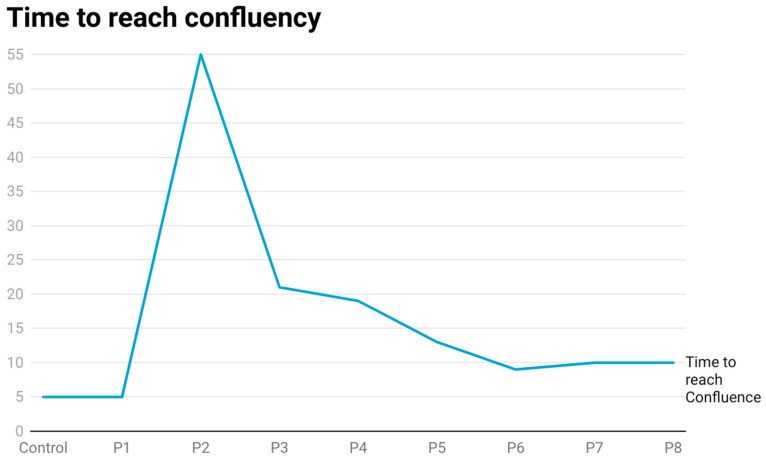
Time to reach confluency for cells treated with cisplatin.

The HRTPT cells were exposed to CisPt for 8 serial passages. The experiment was initiated by exposing confluent HRTPT cells to 1.5 µM CisPt for 48 h before being sub-cultured at a 1:3 ratio into fresh growth media containing the drug. The dose used for this study was based upon the findings of a study where the investigators found that patients treated with CisPt had levels of the drug between 142 and 2.99 × 10^3^ ng/L in their plasma 8–75 months following treatment [13]. At this first passage, the cells were designated as P1. These P1 cells were fed with fresh growth media containing 1.5 µM CisPt every 3 days until confluent. Control HRTPT cells were grown under identical conditions without 1.5 µM CisPt in the growth media. Light microscopic examination of the cultures showed that both the control and the treated cells reached confluency by 5 days and formed domes by day 5 and displayed a similar morphology (Figure 1A,B). The confluent P1 HRTPT cells exposed to CisPt were sub-cultured under identical conditions (P2) but showed a different outcome compared to P1 cells. While the sub-cultured CisPt-exposed cells attached to the flask, they were slow to initiate division and cell spreading at 24 h after sub-culture (Figure 1C). Subsequent light microscopic examination of the CisPt-exposed cells at P2 showed that the cells remained attached but with a very low rate of cell division, as shown for cells at day 7 (D7) and at confluency at day 55 (D55) (Figure 1D,E). While the morphology was similar to control cells, the CisPt-exposed cells did not form domes at confluency. The CisPt-exposed cells at P2 were then serially sub-cultured at confluence for six additional passages. The morphology of the confluent CisPt-exposed cells at P8 was similar to the control cells (Figure 1A,F) but lacked dome formation. The length of time necessary for the CisPt-exposed cells to reach confluency decreased at every passage number until they were near the control cells at P8 (Figure 2).

The confluent CisPt-exposed cells were also examined for their expression of *KIM-1*, *CK-7*, *CK-19*, *MATE1*, *NRF2*, and *HMOX1* genes at P2, P4, P6, and P8 (Figure 1G–L). The results showed that the expression of KIM-1 increased as the passage number increased compared to the control. The expressions of *CK-7*, *CK-19*, and *HMOX-1* were elevated at all passages compared to the control, but with no clear relationship to passage number. The expression of *MATE1* was reduced compared to the control at P2, remained the same at P4, but increased at P6 and P8. The expression of *NRF2* was elevated at all passages with no clear relationship to passage number. The expression of *PROM1* and *CD24* was determined on confluent HRTPT cells exposed to CisPt at passages 2, 4, 6, and 8. The expression of *PROM1* was decreased or remained the same in all passages compared to the control (Figure 1M). The expression of *CD24* remained the same at P2 and P6 and showed an increase at P4 and P8 when compared to the controls (Figure 1N). The accumulation of Pt in the CisPt-exposed HRTPT cells was also determined for confluent cells at P2, P4, P6, and P8 (Figure 1O). There was Pt accumulation at all four passages, but P2 and P4 had the lowest accumulation, while P6 and P8 were the highest, with both having a similar accumulation.

### 2.2. Alterations in Global Gene Expression for Control HRTPT Cells and HRTPT Cells Exposed to 1.5 µM CisPt for 8 Passages

**Figure 3:**
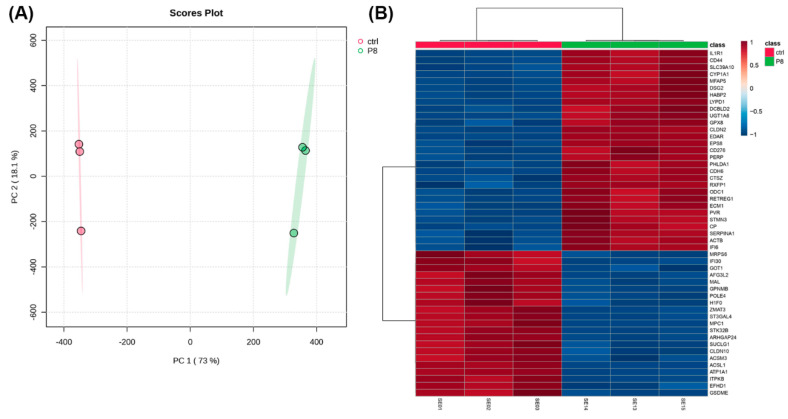
Heatmap and PCA analysis of HRTPT cells exposed to 1.5 µM cisplatin. (**A**) The PCA score plot displays the results of a principal component analysis (PCA) performed on the RNA sequencing dataset. The plot shows the distribution of different samples across the first two principal components, which account for 73% and 18.1% of the variance, respectively. Labels on the points denote individual sample IDs. (**B**) Heatmap of average gene expression for the top 50 differentially expressed genes identified through ANOVA analysis for the different passage conditions. The color bar shows the average expression level.

RNA-seq was performed to determine the differential gene expression between control HRTPT cells and those exposed to 1.5 µM CisPt for eight passages. Principal component analysis (PCA) demonstrated an unbiased distribution of the triplicate determinations for both the control and treatment groups (Figure 3A). The heatmap shows the magnitude of individual values between the two groups (Figure 3B).

**Figure 4:**
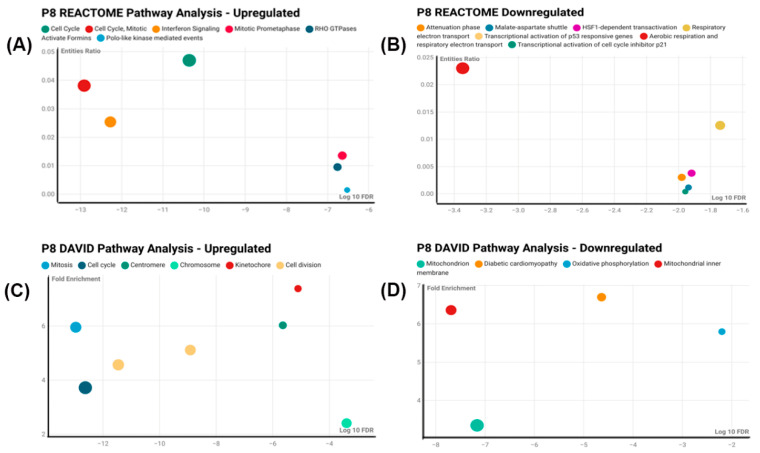
Pathway analysis with DAVID and REACTOME: The scatter plot displays pathways upregulated and downregulated at P8 as analyzed using DAVID and REACTOME. The vertical axis represents the fold enrichment/entities ratio, while the horizontal axis shows the -Log10 false discovery rate (FDR). Each bubble corresponds to a specific pathway, with the size of the bubble indicating the count of genes involved in that pathway. Larger bubbles represent pathways with a greater number of associated genes.

The RNA-seq results demonstrated that 516 genes were differentially expressed between the control cells and cells that had been treated with 1.5 µM CisPt for eight passages (Table S1). Of these, 354 were upregulated and 162 were downregulated compared to the control cells (Table S1). The differentially expressed genes were evaluated for their enrichment and identification with processes and pathways using several public software programs. The analysis of the total 516 differentially expressed genes by Reactome [14] demonstrated processes associated with cell division, actin cytoskeleton, and extracellular matrix (Table S2). The differential genes that were upregulated followed the above pattern, which would also be associated with promoting cell division (Figure 4A). The downregulated genes were related to survival, repair processes, and mitochondrial-mediated apoptosis (Figure 4B). The downregulated genes were fewer in number and had lower *p*-values and FDRs (Table S2). Real-time reverse transcription PCR analysis of the genes FN1, CDH6, TUBA1B, TUBB, DEPTOR, and SLC4A4 showed a correlation to the findings of the RNA-seq data (Figure S1). These genes were chosen based on their involvement in cell cycling, renal damage, repair, and transport processes. An analysis of the differentially expressed genes using the DAVID analysis program [15,16] demonstrated enrichment of genes associated with cell division for both the combined set of up- and downregulated genes and the upregulated genes alone (Table S2). Genes associated with the extracellular matrix were also clustered for both the up- and downregulated genes and the upregulated alone. For the upregulated genes, they followed the above pattern, which would also be associated with promoting cell division (Figure 4C). For the downregulated genes, the two clusters with the highest enrichment scores were identified with mitochondrial function (Figure 4D). A pathway analysis using Panther [17] was informative only for the combined gene set and identified p53 and integrin binding as pathways (Table S2). Overall, cell growth and cellular matrix were the most common features identified using the three analysis programs.

The possible relationship between the differentially expressed genes was also determined in comparison to markers used to define the nephron segments of the normal kidney. The nephron markers used for this study were identified from the existing literature and new studies [18]. The results showed that there were no differentially expressed genes found among 18 marker genes defining the proximal tubule; 5 markers genes for the macula densa; 14 marker genes for the cortical collecting, outer medullary, and inner medullary collecting duct; and 28 marker genes for papillary epithelial cells, intercalated cell, cortical collecting duct, intercalated cell type A, connecting tubule intercalated cell type A, outer medullary collecting duct intercalated cell type A, and intercalated beta cell (Table S3). The positive correlations were 4 out of 21 genes for the thin limb (*CLDN1*, *CLDN10*, *ID1*, *JAG1*); 2 out of 20 for the thick ascending limb (*CYFIP2*, *RAP1GAP*); 1 out of 20 for the distal convoluted tubule (*ITPKB*); and 1 out of 15 for the connecting tubule and principal cell (Table S3).

**Figure 5:**
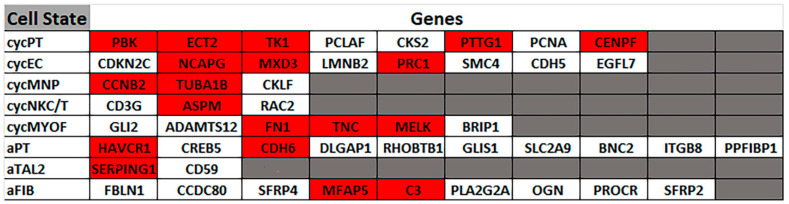
CisPt-treated HRTPT kidney progenitor cells upregulate genes associated with cycling and adaptive/maladaptive/repair states. Clustergram showing the correlation of the upregulated genes in progenitor cells treated with CisPt P8 with known gene markers of various kidney cell types identified in acutely and chronically injured human kidneys, including, cycling proximal tubular cells (cycPT), cycling mononuclear polymorph cells (cycMNP), cycling endothelial cells (cycEC), cycling natural killer and T cells (cycNKC/T), cyclic myofibroblast (cycMYOF), adaptive proximal tubular cells (aPT), adaptive thick ascending limb cells (aTAL2), and adaptive fibroblasts (aFIB). The cell states are arranged in the first column and genes are arranged in rows with the positively corelated genes in red color. The genes that do not correlate are indicated by the white color.

Furthermore, a positive correlation analysis of the upregulated genes in cells treated with CisPt for eight passages revealed a unique set of gene markers identified previously in the Human Kidney Atlas Project [18] using both snRNA-seq and scRNA-seq platforms (Figure 5). These markers uniquely define distinctly altered cell expression profiles that deviate from expression levels in normal reference cells and appear only in acute and chronic kidney injury. This altered status includes several categories, namely, adaptive/maladaptive/repairing, which includes cells that retain differentiation markers of reference states at lower levels and express known injury-associated genes, mesenchymal markers, or factors promoting inflammation or fibrosis; degenerative state, which includes cells characterized by a marked loss of differentiation markers, increased expression of endoplasmic reticulum and mitochondrial genes, and/or a significant decrease in gene detection, and may represent an early injury state or cells that will not recover function; and cycling cell status, which includes cells enriched with cell cycle genes, indicating a reparative/regenerative process. Interestingly, cells treated with CisPt for eight passages significantly upregulated 5 out of 8 genes (*PTTG1*, *CENPF*, *TK1*, *PBK*, *ECT2*) that characterize the cycling proximal tubular cells, 3 out of 8 genes (*NCAPG*, *PRC1*, *MXD3*) that characterize the cycling endothelial cells, 3 out of 6 genes (*MELK*, *TNC*, *FN1*) that characterize the cycling myofibroblasts, 1 out of 2 genes *(SERPING1*) that characterize the adaptive thick ascending limb cells, 2 out of 10 genes (*HAVCR1*, *CDH6*) that characterize the adaptive proximal tubular cells, and 2 out of 11 genes (*MFAB5*, *C3*) that characterize the adaptive fibroblasts. These results support our previous Reactome analysis, showing enrichment of cell cycle genes and indicating that our cells are proliferative and overcome the cell cycle arrest induced by cisplatin. It also indicates an active adaptive process in these cells. 

**Figure 6:**
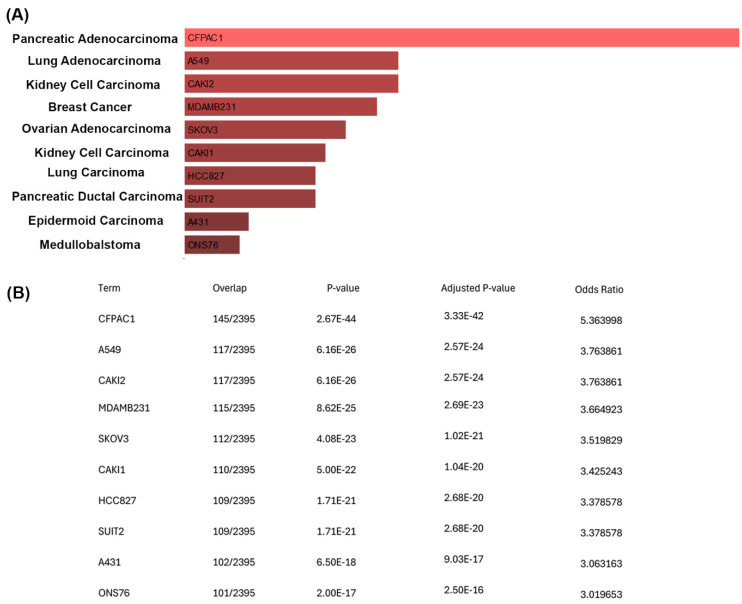
Chronic CisPt treatment in progenitor cells upregulates genes enriched in cancer cells. (**A**) Bar graph demonstrating the correlation between differentially expressed genes in CisPt-treated progenitor cells at P8 and cancer cells identified in ARCHS4 cell line datasets. The length of each bar represents the significance of a specific gene set. Additionally, the brighter the color, the more significant the gene set is. (**B**) A raw data table showing the ratio of differentially expressed genes in CisPt-treated progenitor cells that overlap with the enriched genes of each cancer cell line identified in the ARCHS4 dataset, along with the *p*-value, adjusted *p*-value, and odds ratio.

We performed a positive correlation analysis between the upregulated genes in cells treated with CisPt for eight passages and the ARCHS4 cell line database using Enricher [19,20,21,22]. Strikingly, out of the 354 upregulated genes in cells treated with CisPt for eight passages, 145 genes matched the 2395 genes that are enriched in the CFPAC1 pancreatic adenocarcinoma cell line. Additionally, 117 genes matched the 2395 genes that are enriched in the A549 lung adenocarcinoma cell line, 117 genes matched the 2395 genes that are enriched in the CAKI2 kidney clear cell carcinoma cell line, 115 genes matched the 2395 genes that are enriched in the MDA-MB-231 breast cancer cell line, 112 genes matched the 2395 that are enriched in the SKOV3 ovarian adenocarcinoma cell line, and 110 genes matched the 2395 genes that are enriched in the CAKI1 clear cell kidney carcinoma cell line (Figure 6).

## 3. Discussion

There are limited studies examining the effect of non-lethal levels of CisPt on putative cultures of human proximal tubule cells or human renal cells co-expressing PROM1 and CD24 following serial passage of exposed cells. As detailed in the introduction, this laboratory isolated a renal epithelial cell line, HRTPT, exclusively co-expressing PROM1 and CD24, which displayed features expected of a renal epithelial progenitor cell. As detailed [23] clinical studies on patients treated with CisPt as a chemotherapeutic agent show, there is a high prevalence of nephrotoxicity, which occurs in approximately 30% of patients. Nephrotoxicity is most often seen after 10 days of treatment, depending on the dose, and is characterized by a reduced glomerular filtration rate, higher serum creatinine, and reduced serum magnesium and potassium levels. The most severe and common feature of CisPt nephrotoxicity is acute kidney injury (AKI), often referred to as acute tubular necrosis (ATN). ATN can be resolved after treatment or progress to chronic kidney disease (CKD). However, factors favoring progression or resolution, along with the possible long-term effects of CisPt on renal function, remain an area of investigation.

The capacity of the kidneys to regenerate functional tubules during and following tubule damage from AKI has been known for decades [24,25,26,27]. A vital component of this repair mechanism is human adult renal progenitor/stem cells, which can replace terminally damaged cells through proliferation and differentiation into various renal cell types [28]. The present study was designed to determine the effect of CisPt at a non-lethal dose on HRTPT renal progenitor cells over eight serial passages. Confluent HRTPT cells exposed to 1.5 µM CisPt for 48 h and then sub-cultured in CisPt-containing growth medium showed no overt toxicity compared to controls when examined by light microscopy. However, when P1 cells were sub-cultured at a 1:3 ratio under identical conditions, the cells attached to the culture surface, but there was no evidence of proliferation. The first three days of feeding of fresh growth media did not remove the CisPt-exposed cells from the growth surface. It was decided to keep feeding the P2 cells every three days and monitor any evidence of cell growth. As detailed in the results, the CisPt-exposed cells grew very slowly and finally proliferated to confluency. These data suggest that HRTPT cells can survive the growth arrest elicited by exposure to CisPt. Subsequent sub-culture of the cells showed that the time to confluency decreased sequentially until at P8, where the time to confluency and morphology of the treated cells was similar to control cells. The only marked morphological difference between control and P8 cells was that the cells at P8 showed no evidence of dome formation. A surprising finding was that the level of Pt within the cells increased through passage six and remained at that level through passage eight. These results show that CisPt-exposed progenitor cells can overcome growth arrest and regain proliferation while maintaining a substantial intracellular level of Pt. It has been reported that renal progenitor cells are resistant to CisPt [27,28,29]. The current finding would be consistent with resistance to CisPt, but with the caveat that progenitor cells are able to maintain an intracellular level of Pt despite a normal light level morphology and growth. The cells at P8 also expressed markers of cell damage and stress when compared to the untreated control cells. Overall, these observations suggest that the progenitor cells exposed to CisPt through P8 may represent a progenitor cell with an altered phenotype that can participate in renal repair and regeneration. Of importance is that the CisPt-exposed cells at P8 retain the progenitor markers PROM1 and CD24, although at a lower level.

Since CisPt-exposed cells at P8 had several differences compared to control cells, an RNA-seq analysis was performed to define the differential gene expression between the two cell populations. A total of 516 genes were differentially expressed, of which 354 were upregulated and 162 were downregulated compared to the control cells. The first indication that the differentially expressed genes might have a relationship with the cell cycle was suggested by using Reactome, DAVID, and Panther analysis programs.

Next, it was determined if the differentially expressed genes correlated to gene markers commonly used by others to define distinct cellular elements of the normal human nephron. This analysis showed no correlation for most nephron cell types, with only the thin limb (4 of 21 genes) and the thick ascending limb (2 of 20 genes) having more than one marker gene in common for a nephron cell type. This effectively showed that the progenitor cells treated with CisPt displayed no identity, or close identity, with cells of the normal human nephron. This study was extended to determine if any of the differentially expressed genes correlated with cells associated with a damaged nephron. This analysis showed a strong correlation with genes involved with cell cycling and adaptive status. Cycling status was associated with the proximal tubule, endothelium, and myofibroblast cells, while adaptive processes were associated with the thick ascending limb, proximal tubule, and fibroblasts. This indicates that CisPt-containing renal progenitor cells can potentially participate in repair and regeneration. The functional properties of the cell regenerated from CisPt-generated progenitor cells require further investigation.

The current study exposed progenitor cells to CisPt for eight serial passages or approximately 12 population doublings at a non-lethal level of exposure. This length of exposure raises the question as to the length of time altered progenitor cells might be present in a treated patient. Clinical studies on cancer patients treated with CisPt suggest intracellular levels of Pt might persist long after the conclusion of treatment. In a study of 45 patients, 8–75 months after treatment with CisPt, the plasma concentration of the drug ranged between 142 and 2.99 × 10^3^ ng/L in individuals [13]. Twenty-four percent of plasma Pt was recovered in the ultrafiltrate, and up to 10% showed reactivity. Pt levels were related to time, dose, and glomerular filtration rates (GFR). The author’s proposed that Pt in plasma represents Pt eliminated from regenerating tissue. An additional study performed a 20-year follow-up on 458 survivors of testicular cancer who were treated with Pt-based chemotherapy [30]. The median observation time was 20 (range = 13–28) years, and the median plasma Pt level according to the treatment group was surgery, 50 ng/L; cisplatin ≤850 mg, 85 ng/L; cisplatin > 850 mg, 106 ng/L; and carboplatin, 40 ng/L. This study demonstrated that circulating Pt is still detectable in plasma up to 28 years after cisplatin-based chemotherapy. Pt levels were significantly associated with previous cisplatin therapy with respect to both dose and time from treatment. These findings support the concept that CisPt could linger in renal progenitor cells after patient recovery from acute injury and participate in the progression to chronic kidney disease. This also might explain the long-term renal abnormalities thought to occur after Pt-based chemotherapy.

To further understand the growth of the CisPt-exposed progenitor cells at P8, a correlation analysis was performed between the upregulated genes and the ARCHS4 cell line database using Enricher [19,20,21,22]. This analysis showed a striking number of upregulated genes that were also expressed in cancer cell lines from different types of cancer. Of note were the 110 upregulated genes that matched the 2395 genes that are enriched in the CAKI1 clear cell kidney carcinoma cell line. However, there is no clinical evidence that cancer patients treated with CisPt have an increased risk of developing secondary cancers [31]. This conclusion was based on 28 trials with 7403 patients with second cancers noted in 143 patients, with 75 in the CisPt arm and 68 in the non-CisPt arm. It is likely that the CisPt-exposed cells at P8 correlated with genes associated with various cancer cell lines, simply reflecting their elevation of cell cycle genes associated with the repair or regeneration process. The CisPt-exposed renal progenitor cells showed no loss of contact inhibition of growth, as noted by the absence of any foci of multilayered cells or uneven areas of the monolayer. However, the significance of this correlation requires further study.

A search of the literature identified two other studies that examined the role of human adult renal progenitor cells in CisPt-induced nephrotoxicity [27,32]. In the first study, the authors used cells from two sources: PROM1+ tubular adult renal progenitor cells (tARPC) purified from the healthy parts of kidney cortexes from patients undergoing nephrectomy for renal clear-cell carcinoma, and immortalized human primary renal cells (RPTEC/TERT1) cells that were obtained from a commercial vendor. The investigators performed co-culture experiments where the tARPCs were seeded on top of the trans-well filters and the RPTECs damaged with 2.5 mmol/L cisplatin for six hours were placed on the bottom of the filter after 24 h. The cells were co-cultured for four days, and the results of this study showed that co-culture with tARPCs significantly reduced the damage to the RPTECs elicited by previous exposure to CisPt. Further studies showed that there was a decrease in necrosis and apoptosis in the damaged RPTECs, and genes were identified that were associated with this reduced damage. While this study used high levels of CisPt exposure and a shorter time course than the present study, it does suggest that undamaged renal pro-genitor cells can release factors into the growth media that reduce the damage to cells previously injured with CisPt. In the second study [32], the same group of authors showed that the repair induced by tARPCs occurs by the activation of toll-like receptor 2 and was mediated by the secretion of inhibin-A and decorin directly as proteins or as microvesicles containing mRNAs. An interesting question not addressed is the possibility that the RPTEC cells also express a high percentage of cells that co-express PROM1 and CD24 [12,33]. This raises the possibility that the action on the damaged cells may have increased the response of existing renal progenitor cells to reduce the toxicity of the CisPt-induced damage. The likelihood that the RPTECs possess a prominent population of progenitor cells is suggested since PROM1 was not an upregulated gene in the -omic analysis. This might indicate a relationship based on secreted factors between progenitor cells in damaged and undamaged cells.

In conclusion, our study demonstrates that renal progenitor cells can recover from CisPt exposure and regain proliferative potential in the continued presence of both extracellular CisPt and intracellular Pt. The recovered cells correlate to the cycling and adaptive status of damaged renal cells likely involved in repair and regeneration.

## 4. Materials and Methods

### 4.1. Cell Culture

The RPTEC/TERT1 cells were obtained from the American Type Culture Collection and were grown using serum-free conditions as previously described by this laboratory [34,35]. The growth formulation consisted of a 1:1 mixture of Dulbecco’s Modified Eagle’s Medium (Gibco, Thermo Fisher Scientific, Waltham, MA, USA) and Ham’s F-12 growth medium supplemented with selenium (5 ng/mL), insulin (5 µg/mL), and transferrin (5 µg/mL) (Corning Incorporated, Corning, NY, USA), hydrocortisone (36 ng/mL) (Thermo Fisher Scientific, Waltham, MA, USA), triiodothyronine (4 pg/mL) (Thermo Fisher Scientific, Waltham, MA, USA), and epidermal growth factor (10 ng/mL) (Gibco, Thermo Fisher Scientific, Waltham, MA, USA). In a previous study [12], confluent cultures of the immortalized RPTEC/TERT cell line were sorted into two different cell populations, namely, HRTPT (CD133+/CD24+) cells and HREC24T (CD133−/CD24+) cells, using BD FACSAria (BD Biosciences, Franklin Lakes, NJ, USA). In this study, we used the previously isolated HRTPT cells, which were seeded at a density of 1,700,000 cells in a T-75cm^2^ flask and allowed to reach confluency before being exposed to CisPt (Selleck Chemicals, Houston, TX, USA) and dissolved in saline. For long-term exposure, the HRTPT cells were grown in the presence of 1.5 µM CisPt for eight serial passages. This dose was based upon the findings [13] in a study where they found that patients treated with CisPt had levels of the drug between 142 and 2.99 × 10^3^ ng/L in their plasma 8–75 months following treatment. The cells were scraped in phosphate-buffered saline (PBS) from Fisher Scientific, Hampton, NH, USA, following treatment, washed, and centrifuged to obtain pellets, which were stored at −80 °C until further analysis. All experiments were performed in triplicate.

### 4.2. Cell Viability Assay

Twenty-five hundred cells were seeded into a 96-well plate from Corning, Corning, NY, USA. Upon reaching confluency and dome formation, cells were treated with cisplatin (Selleck Chemicals, Houston, TX, USA) and allowed to grow for 72 h. After this period, the culture medium was discarded, and the plate was incubated with 2.5% crystal violet (Sigma-Aldrich, St. Louis, MO, USA, dissolved in 20% methanol from Fisher Scientific, Hampton, NH, USA) while being agitated slowly for 15 min. The plates were washed with double-distilled water (ddH_2_O) and allowed to air dry overnight. The cells were then lysed with 0.1 M sodium citrate in 25% ethanol (Thermo Fisher Scientific, Waltham, MA, USA) (pH 4.2), and absorbance was measured at 570 nm using a microplate spectrophotometer (BioTek EL800, Santa Clara, CA, USA). The data of the preliminary toxicity study are shown in Figure S2.

### 4.3. ICP-MS

For ICP-MS analysis, cell pellets were digested in nitric acid (HNO_3_) using a microwave digestion system (Milestone, Sorisole, Bergamo, Italy). The digestion protocol involved adding 1 mL of concentrated HNO_3_ (Thermo Fisher Scientific, Waltham, MA, USA) to each pellet, followed by microwave digestion at 180 °C for 30 min. After digestion, the samples were diluted to a final volume of 10 mL with ultrapure water (MilliporeSigma, Burlington, MA, USA). The diluted samples were then filtered through a 0.22 µm filter (MilliporeSigma, Burlington, MA, USA) to remove any particulate matter.

The ICP-MS system (Thermo Scientific iCAP Qc, Thermo Fisher Scientific, Waltham, MA, USA) was calibrated using a series of standard solutions containing known concentrations of platinum. Quality control samples and blanks were included in each batch of analyses to ensure accuracy and precision. The digested samples were analyzed for platinum content, and the data were quantified based on the calibration curve obtained from the standards.

#### ICP-MS Data Analysis

The ICP-MS data were processed using Qtegra software Version 2.2 (Thermo Fisher Scientific, Waltham, MA, USA). The concentrations of platinum in the cell pellets were normalized according to the weight of the pellets. Statistical analysis was performed using GraphPad Prism 9.1.1 (GraphPad Software, San Diego, CA, USA), and significance was determined by ANOVA followed by Tukey’s post-hoc test, with *p* < 0.05 considered statistically significant.

### 4.4. Real-Time PCR Analysis

The level of expression of PROM1, CD24, keratin 7 (*CK7*), keratin 19 (*CK19*), heme-oxygenase 1 (*HMOX1*), nuclear factor erythroid 2-related factor 2 (NrF2), multidrug and toxin extrusion protein 1 (*MATE1*), kidney injury molecule-1 (KIM-1), fibronectin1 (FN1), cadherin 6 (CDH6), tubulin alpha 1b (T*UBA1B*), tubulin beta class 1 (*TUBB*), DEP domain-containing mTOR-interacting protein (*DEPTOR*), and solute carrier family 4 member 4 (*SLC4A4*) genes was assessed using real-time reverse transcription PCR and commercially available primers. The primers for *PROM1*, *CK24*, *CK7*, *CK19*, *HMOX1*, *NRF2*, *MATE1*, *KIM-1*, *FN1*, *CDH6*, *TUBA1B*, *TUBB*, *DEPTOR*, and *SLC4A4* were obtained from IDT (Integrated DNA Technologies, Coralville, IA, USA). A quantity of 1 µg of cDNA was synthesized from the total RNA using the LunaScript^®^ RT SuperMix Kit (New England Biolabs #E3010L, Ipswich, MA, USA) per the manufacturer’s protocol. cDNA was diluted with nuclease-free water to achieve a final concentration of 10 ng/µL.

A quantity of 2 µL of cDNA (20 ng) was used in a 20 µL qPCR reaction and analyzed using the BioRad CFX96 Touch Real-Time PCR Detection System (Hercules, CA, USA) and the Luna^®^ Universal qPCR Master Mix (New England Biolabs # M3003E). qPCR cycle conditions were one cycle of 2 min at 95 °C, 40 cycles of 5 s at 95 °C, and 30 s at the annealing temperature of 60 °C. Expression levels were determined from the values of threshold cycle (Ct) using the method of 2^−ΔΔCt^ and using β-actin or 18s as the reference control genes.

### 4.5. RNA Sequencing

#### 4.5.1. RNA Extraction

Total RNA was extracted from HRTPT cells using the RNeasy Mini Kit (Qiagen, Hilden, Germany), following the manufacturer’s protocol. Triplicate samples were prepared and diluted to a concentration of 100 ng/mL. The RNA samples were packed in dry ice and transported to Columbia University for further processing.

#### 4.5.2. Library Preparation

RNA sequencing libraries were prepared using the Illumina TruSeq Total RNA Kit (Illumina, San Diego, CA, USA) according to the manufacturer’s instructions. The Illumina TruSeq PCR reaction was modified by using KAPA HiFi HotStart Ready Mix (Kapa Biosystems, Wilmington, MA, USA) for the final PCR amplification step, which was adapted to fit the Aviti workflow. The prepared libraries were sequenced on the Element Aviti platform (Element Biosciences, San Diego, CA, USA), generating approximately 40 million paired-end reads of 75 base pairs in length.

#### 4.5.3. Data Analysis

RNA-seq raw reads were stored in FASTQ format files containing the sequence data. FastQC tool (version 0.12.1, Babraham Research Campus, Cambridge, UK) was used to assess the quality of raw sequencing data. As per the quality control report on the raw data, files were subjected to pre-processing using the fastp tool (version 0.17.0, Shenzhen Institutes of Advanced Technology, Chinese Academy of Sciences, Shenzhen, China, accessed on 21 November 2024) to trim low-quality bases and adapter sequences. After trimming, alignment of the reads to a reference genome was performed using STAR (version 2.7.10b, Cold Spring Harbor Laboratory, Cold Spring Harbor, NY, USA, and Pacific Biosciences, Menlo Park, CA, USA, accessed on 21 November 2024). A count matrix was generated using FeatureCounts (version 2.0.7, The University of Melbourne, Parkville, Victoria 3010, Australia, accessed on 21 November 2024) from the Subread package. The normalized distribution of the counts was checked to identify the outliers. The median-of-ratios method was applied from the DESeq2 package (version 1.46.0, European Molecular Biology Laboratory, Heidelberg, Germany, accessed on 21 November 2024), to perform differential expression testing. When comparing the groups, the statistically significant genes that were differentially expressed in the sub-selections of the samples were identified using a two-tailed Student’s *t*-test. The significance level of the *p*-value < 0.05 was employed as a standard to filter genes. Principal component analysis (PCA) was performed on the RNA-seq to describe the relationship between the samples by visualizing the samples and groups in two-dimensional space. The goal here was to determine the distribution of samples and visualize how the global gene expression profile is scattered in different groups. Pearson correlation was used to find the relationship between the different passage conditions as well as genes, and the heatmap method was used to plot the correlation coefficient value to find the most correlated samples. The entire analysis was performed using R (version 4.3.2, R Foundation for Statistical Computing, Vienna, Austria, accessed on 21 November 2024) and Bioconductor (version 3.20).

### 4.6. Statistical Analysis

All experiments were performed in triplicate, and the data were analyzed using ANOVA with Tukey post hoc testing performed by GraphPad Prism 9.1.1 (GraphPad Software, San Diego, CA, USA). The data are plotted as the mean ± SEM of triplicate determinations.

## Data Availability

Dataset available on request from the authors.

## Supplementary Materials

The following supporting information can be downloaded at: https://www.mdpi.com/article/10.3390/ijms252312553/s1.
