# Supplementary figures and images for: Effect of Long-Term Cisplatin Exposure on the Proliferative Potential of Immortalized Renal Progenitor Cells
Source: Int J Mol Sci. 2024 Nov 22;25(23):12553. doi: 10.3390/ijms252312553 (PMC11640886; doi:10.3390/ijms252312553)

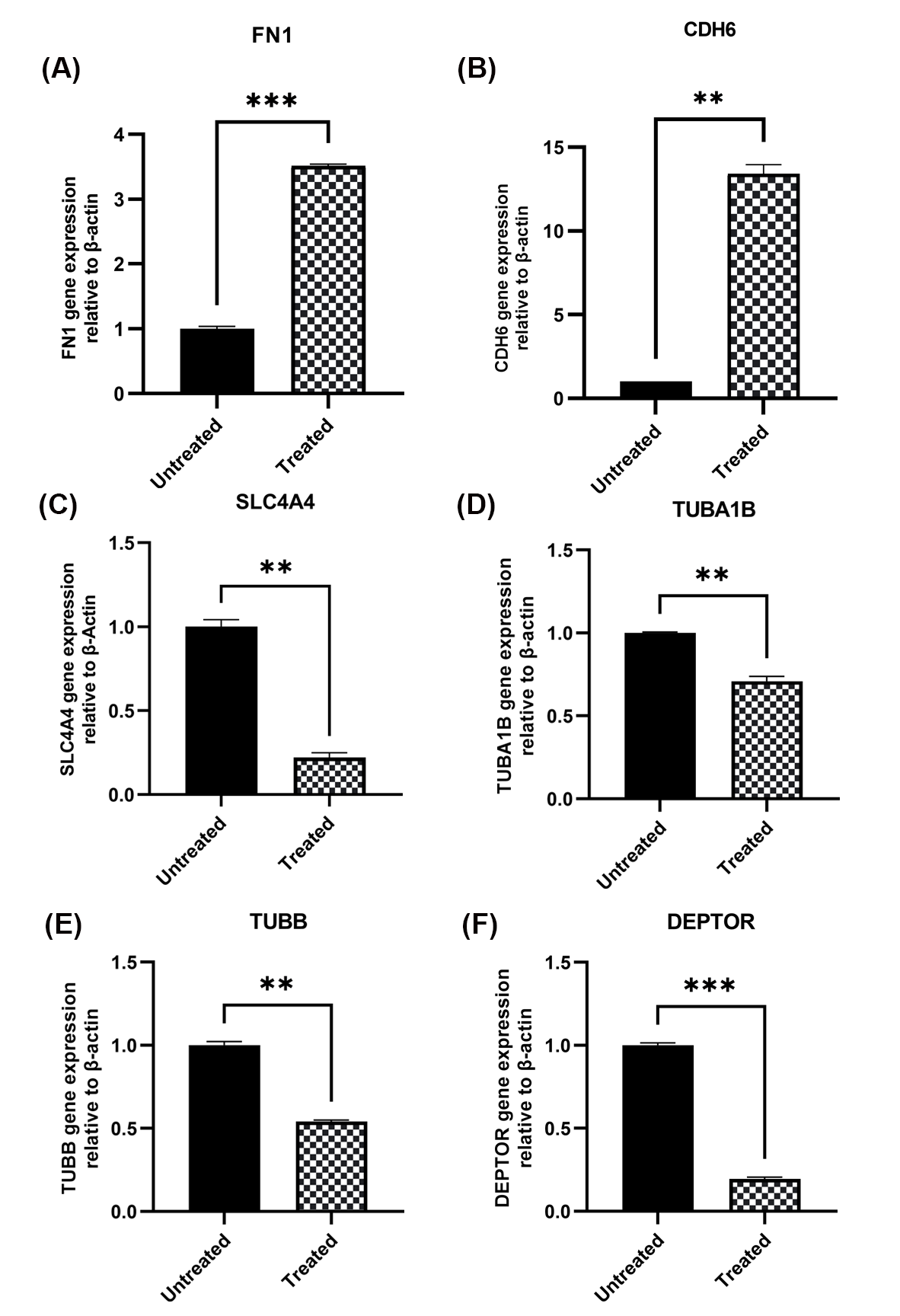

Supplement: Supplementary file 1 [file ijms-25-12553-s001.zip › Fig S1.tif]

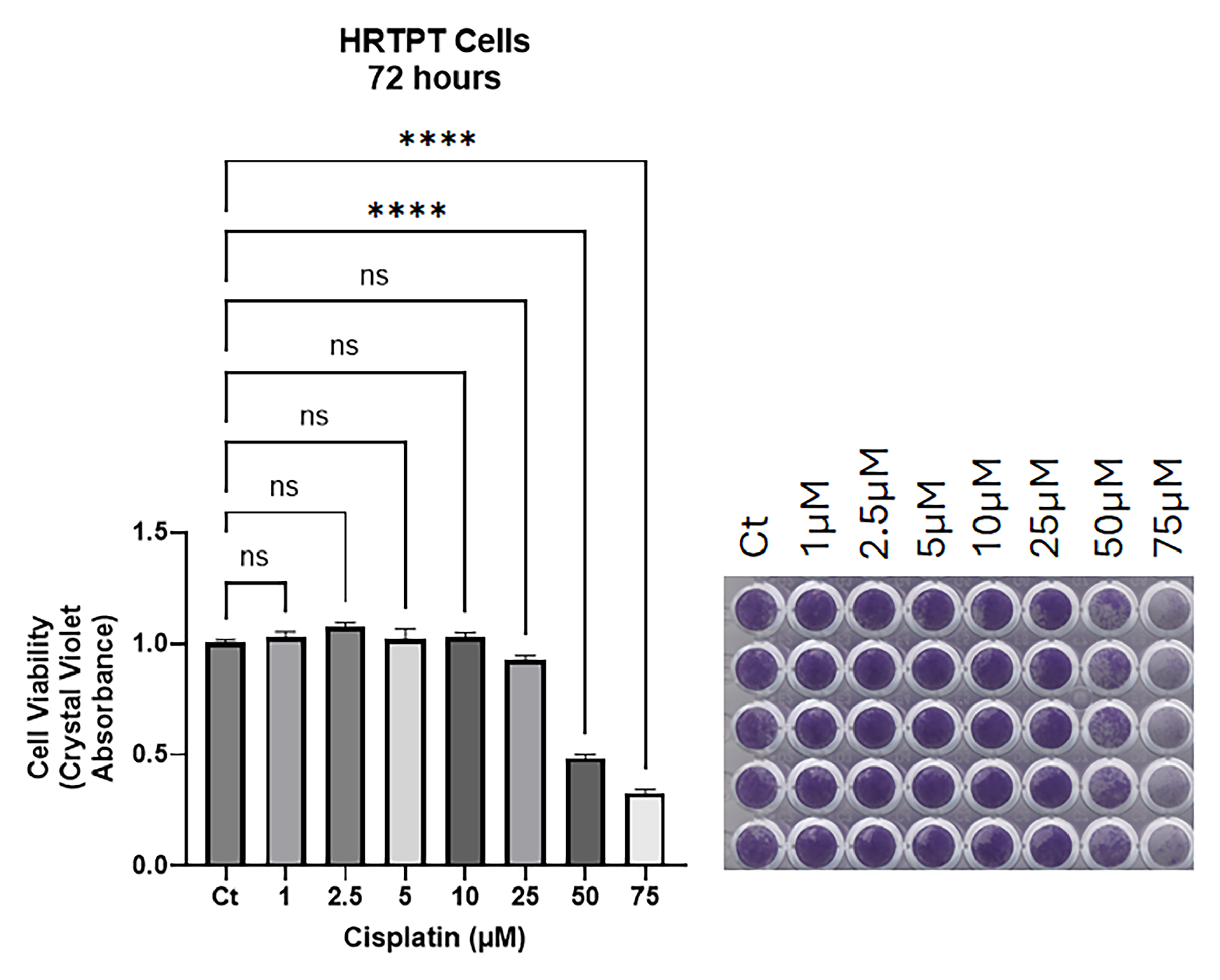

Supplement: Supplementary file 1 [file ijms-25-12553-s001.zip › Figure S2 cisplatin toxicity.tif]
